# Diversity and Influencing Factors of Endosymbiotic Bacteria in *Tetranychus truncatus* Sourced from Major Crops in Xinjiang

**DOI:** 10.3390/insects16111126

**Published:** 2025-11-04

**Authors:** Kaiqin Mu, Bing Zhang, Zhiping Cai, Jing Chen, Jianping Zhang, Jie Su

**Affiliations:** Key Laboratory of Oasis Agricultural Pest Management and Plant Protection Resources Utilization, College of Agriculture, Shihezi University, Xinjiang, Shihezi 83200, China

**Keywords:** *Tetranychus truncatus*, endosymbiotic bacteria, host plant, environmental factor

## Abstract

**Simple Summary:**

*Tetranychus truncatus* exhibits rapid reproduction and a broad host range, posing a significant threat to major crops in Xinjiang, such as cotton, corn, and soybeans. Understanding the relationships between host plant species and the composition of endosymbiotic bacterial communities is essential for developing effective strategies. This study systematically investigated the diversity of endosymbiotic bacteria associated with *T. truncatus* collected from three primary crops in Xinjiang. The research results indicate endosymbiotic bacterial diversity in *T. truncatus* populations on corn was significantly higher than that observed in populations on cotton and soybean. The diversity of endosymbiotic bacteria in *T. truncatus* was significantly higher in southern Xinjiang than in northern Xinjiang.

**Abstract:**

The Xinjiang Uygur Autonomous Region, situated in northwest China, boasts a unique geographical position and a consequent variety of environmental characteristics. *T. truncatus* is prevalent throughout this region as the primary pest affecting various crops. In this study, we analyzed the microbial community structures of endosymbiotic bacteria in *T. truncatus* collected from 17 regions and three host plants in Xinjiang using 16S rRNA sequencing. Through composition analysis of the endosymbiotic bacteria in *T. truncatus* from Xinjiang, it was found that the dominant bacterial phyla were Pseudomonadota and Bacillota. At the genus level, in addition to *Wolbachia*, *Cardinium*, and *Spiroplasma* (common symbiotic bacteria in *T. truncatus*), the infection rate of *Rickettsia* in *T. truncatus* in Xinjiang was found to be 92.8%. The diversity of the endosymbiotic bacteria community in *T. truncatus* is shaped by both host plant species and geographical region. Specifically, the endosymbiotic bacterial diversity in *T. truncatus* populations on corn was significantly higher than that observed in populations on cotton and soybean (*p* < 0.05). Furthermore, we discovered the diversity of endosymbiotic bacteria in *T. truncatus* was significantly higher in southern Xinjiang than in northern Xinjiang (*p* < 0.05).

## 1. Introduction

Endosymbiotic bacteria are prevalent across arthropods. Over the course of prolonged evolution, they have established a stable symbiotic relationship with their hosts, where they play a crucial role in regulating their physiological processes [1,2], supplying the host with essential nutrients for growth and development, modulating the host’s reproductive processes and fitness, and assisting the host in resisting adverse environmental conditions [3,4]. For example, *Buchnera aphidicola* in aphids can supply essential amino acids required for epidermal synthesis, while the primary symbiotic bacteria in aphids can provide B vitamins and energy-related metabolites to the host [5,6]. Furthermore, in arthropods, the manipulation of host reproduction by *Wolbachia*, *Spiroplasma*, and *Cardinium* via cytoplasmic incompatibility (CI) has been investigated extensively [7,8]. Although endosymbiotic bacteria are crucial to the adaptability of their host, their community structure can also be readily influenced by various environmental factors, including the host plant, temperature, precipitation, and humidity [9].

Host plants, serving as both the food source and habitat for phytophagous arthropods, exert a significant influence on the endosymbiotic bacterial community within the host [10,11,12], playing a crucial role in shaping the diversity and relative abundance of endosymbiotic bacteria [13]. For instance, the abundance of endosymbiotic bacteria in the Colorado potato beetle reaches its peak when feeding on tomatoes and drops to its lowest level when feeding on eggplants [14]. In addition, the titer of *Wolbachia* was significantly higher when *Tetranychus urticae* fed on soybeans and eggplants, whereas the titer of *Cardinium* was markedly elevated when it fed on morning glories [15]. Similarly, when pea aphids feed on different host plants, the composition and infection rates of their endosymbiotic bacterial communities exhibit significant variation [16]. For instance, when feeding on wheat, the titer of *Buchnera aphidicola* is elevated [17], while in contrast, when consuming *Trifolium*, the titer of *Regiella insecticola* increases [18], and when feeding on *Pisum*, the titer of *Serratia symbiotica* becomes more prominent [19].

The types, distribution, and infection rates of arthropod endosymbiotic bacteria differ across various regions, closely associated with the environmental conditions specific to each region [10]. The geographic distribution and infection frequency of endosymbionts in natural populations of the pea aphid appear to be related to the host plant species, temperature, and precipitation. For instance, *Hamiltonella* is present in pea aphid populations in high temperature regions such as Europe and the United States, this bacterium is absent in populations from China and Japan [20]. Specifically, the pea aphid population in central China shows a relatively high abundance of *Serratia* and *Rickettsia*, whereas the infection rates of *Regiella* are notably higher in pea aphid populations in western and eastern China [21]. Insects can adapt to their local environments by modulating the composition of their endosymbiotic bacteria, while environmental factors can also directly influence the host by altering their bacterial community [22]. For instance, under high-temperature conditions, the titer of *Wolbachia* in *Drosophilidae* and *Aedes aegypti* decreases [23]. In conclusion, the environment may also exert indirect effects on infection frequencies by altering the selection pressures that govern the relationship between endosymbionts and their host organisms.

Xinjiang is located in northwest China, and its distinctive geographical position has given rise to climatic features characterized by significant diurnal temperature variations, extended sunshine duration, and scarce precipitation. The Tianshan Mountains divide Xinjiang into southern and northern regions [24]. In Southern Xinjiang, which has a lower latitude than Northern Xinjiang, more solar radiation is received, resulting in higher temperatures. Conversely, the terrain in northern Xinjiang results in greater precipitation, creating an environment characterized by low temperatures and high humidity [25]. Xinjiang boasts extensive territory and abundant land resources, making it a crucial production base for crops such as cotton, soybeans, and corn. However, *T. truncatus* is prevalent in this region and causes significant damage to agricultural production [26]. This species, classified under the order Acariformes, family Tetranychidae, and genus *Tetranychus*, is characterized by its rapid reproduction rate and broad host range [27]. It infests over 60 crops, causing significant reductions in both crop yield and quality [28]. The role of endosymbiotic bacteria in *T. truncatus* remains largely unexplored due to limited systematic research in this field. Therefore, in this study, 16S rRNA high-throughput sequencing was employed to investigate the diversity of endosymbiotic bacterial communities in *T. truncatus* across three major crops in 17 regions of Xinjiang. By examining the relationships between endosymbiotic bacteria in *T. truncatus* and various host plants, geographical factors, and environmental conditions, this study enhances our understanding of the ecological associations of *T. truncatus* populations in the region.

## 2. Materials and Methods

### 2.1. Experimental Populations

Test spider mites: The test samples were collected from 14 regions in Xinjiang, including Altay, Tacheng, Yili, Kashgar, Aksu, Korla, Urumqi, Turpan, Hotan, and Shihezi, between June and August in 2022 and 2023. The host plants were cotton (*Gossypium hirsutum* L.), corn (*Zea mays* L.), and soybean (*Glycine max* L.), with a total of 190 samples representing 17 geographical populations. The climate data used were the average values over the five years from 2019 to 2023, and the meteorological data were obtained from Xihe Energy Big Data Platform (Table 1). Ten male mites from different populations of *T. truncatus* were collected for morphological identification to confirm their species identity. Additionally, female adult mites from each population were collected and preserved in 1.5 mL centrifuge tubes containing 100% ethanol (the distribution of female mites across all sampling sites is summarized in Table 1). These samples were stored at −20 °C until DNA extraction.

### 2.2. DNA Extraction, PCR Amplification, and Sequencing

Extraction of DNA: A single spider mite was selected and placed in a Petri dish containing distilled water for rinsing. We performed three rounds of washing in sterile PBS (pH 7.4) with 0.1% Tween-20, followed by surface sterilization with 70% ethanol for 30 s. The mite was then transferred to the lid of the dish and allowed to dry for 30 s [29]. DNA extraction was performed according to the instructions provided in the Qiagen DNeasy Blood & Tissue Kit, with modifications made as necessary. The V3–V4 region of the 16S rRNA gene was amplified using the universal primers 338F (5′-ACTCCTACGGGAGGCAGCAG-3′) and 806R (5′-GGACTACHVGGGTWTCTAAT-3′). These primers were prepared by combining a 5 μM upstream primer with a downstream primer [30]. The 50 μL PCR reaction system comprised 4 μL DNA template (1 ng/μL), 25 μL Taq DNA polymerase mixture, 1.5 μL of each primer (upstream and downstream), and 18 μL ddH_2_O. The PCR reaction process was as follows: initial denaturation at 95 °C for 3 min; followed by 29 cycles of denaturation at 95 °C for 30 s, annealing at 55 °C for 30 s, and extension at 68 °C for 30 s; final extension at 68 °C for 10 min; and storage at 10 °C. We took 2 μL of the PCR product, mixed with 1 μL Loading Buffer, and performed electrophoresis on 1.5% agarose gel at 60 V for 30 min for visualization and documentation. We analyzed per-site/per-host mean diversity values (normalizing for sample size differences). The amplicon library was paired-end (2 × 250 bp) and sequenced on an Illumina HiSeq 2500 platform (Shanghai Biozeron Co., Ltd., Shanghai, China) using standard protocols [31,32].

### 2.3. Data Analysis

Firstly, Qiime (version 1.91) was used to excise the primer fragments of the sequences and discard the sequences that did not match the primers. The resulting high-quality sequences were subsequently merged using FLASH (version 1.2.7) software and filtered using Qiime (version 1.91) [32]. Operational taxonomic units (OTUs) were clustered from the high-quality sequences at a sequence similarity threshold of 97%. Data analysis in this experiment was performed by calculating the average number of samples per point. The longest sequence within each OTU was selected as the representative sequence. Subsequently, the representative sequences of each group were annotated against the SILVA database using the BLAST (version 2.14.0) method for species identification. The endosymbiotic bacterial community of *T. truncatus* was classified at the OTU level. To determine the significance of differences between groups, the Tukey test and Wallis rank sum test were performed using Mothur (version 1.30.2). The Chao1 index was used to assess alpha diversity and analyzed using R (version 3.3.1) for the multifactorial Kruskal–Wallis test, while Beta diversity was analyzed in Qiime through the Weighted UniFrac distance algorithm [32,33]. The relationship between the flora and environmental factors, as well as among environmental factors, was analyzed using the Mantel test in R (version 3.3.1). Subsequently, the influence of environmental factors on the endosymbiotic bacteria of *T. truncatus* across different hosts at both the phylum and genus levels was evaluated using a correlation heat map [31].

## 3. Results

### 3.1. The Composition of Endosymbiotic Bacterial Communities in Different Populations of T. truncatus in Xinjiang

**Figure 1:**
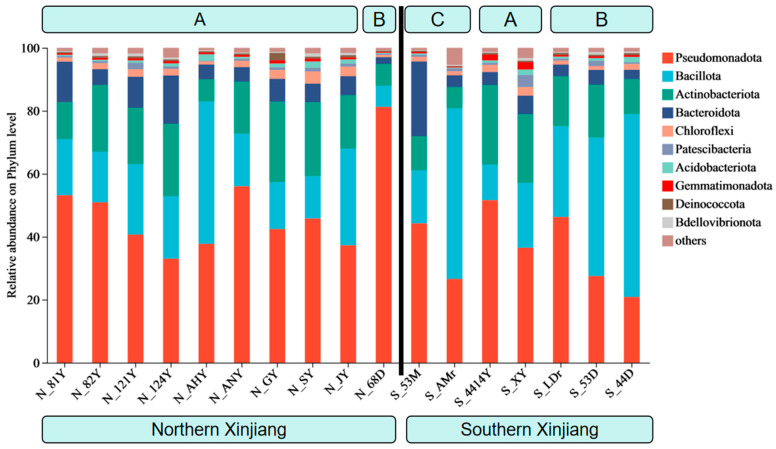
Compositions of symbiotic bacteria in different geographical populations of *T. truncatus* in Xinjiang (phylum level). (Note: A: corn population; B: soybean population; C: cotton population).

In Xinjiang, the phylum-level composition of endosymbiotic bacteria in the different geographical populations of *T. truncatus* is predominantly Pseudomonadota (formerly known as Proteobacteria) and Bacillota (formerly known as Firmicutes), but there are differences across various regions and host plants. In the southern Xinjiang region, the gut microbiota of *T. truncatus* on corn is predominantly composed of Pseudomonadota (36.5–51.6%). In contrast, for *T. truncatus* on soybean, Bacillota is the dominant phylum (28.9–58.1%), followed by Pseudomonadota (20.9–46.2%). On cotton, the dominant bacterial phyla in *T. truncatus* are Pseudomonadota (26.5–44.3%) and Bacillota (16.8–54.2%). In the northern Xinjiang region, Pseudomonadota was the dominant phylum for *T. truncatus* on soybeans (81.3%) and corn (33.0–56.0%) (Figure 1).

**Figure 2:**
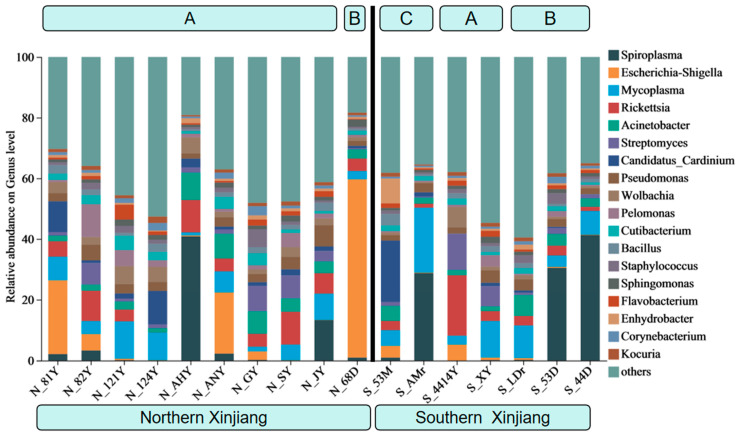
Composition of symbiotic bacteria in different geographical populations of *T. truncatus* in Xinjiang (genus level). (Note: A: corn population; B: soybean population; C: cotton population).

At the genus level, the primary endosymbiotic bacteria associated with *T. truncatus* include *Spiroplasma*, *Escherichia-Shigella*, *Rickettsia*, *Mycoplasma*, *Wolbachia*, and *Cardinium*. In southern Xinjiang, *Rickettsia* (3.3–20.0%) and *Mycoplasma* (2.9–12.0%) are the predominant bacterial genera in *T. truncatus* populations on corn, while *Spiroplasma* (0.2–41.3%) dominates in populations on soybean. On cotton, the dominant bacterial genera include *Spiroplasma* (1.0–28.7%), *Cardinium* (1.4–20.2%), and *Mycoplasma* (5.1–21.4%). In northern Xinjiang, *Escherichia-Shigella* (58.7%) is the predominant genus in *T. truncatus* populations on soybean. However, the dominant bacterial genus in *T. truncatus* populations on corn varies considerably across different regions of northern Xinjiang. Among the samples, *Escherichia-Shigella* was the dominant genus in N_81Y and N_ANY, comprising 24.3% and 20.1%, respectively. In contrast, *Spiroplasma* was the predominant genus in N_AHY and N_JY, accounting for 40.9% and 13.3%. Furthermore, the endosymbiotic bacteria of the two corn varieties, N_SY and S_4414Y, do not include *Spiroplasma* as a component associated with *T. truncatus* (Figure 2).

### 3.2. Analysis of the Alpha Diversity of Endosymbiotic Bacteria Communities in T. truncatus from Different Geographical Populations in Xinjiang

**Figure 3:**
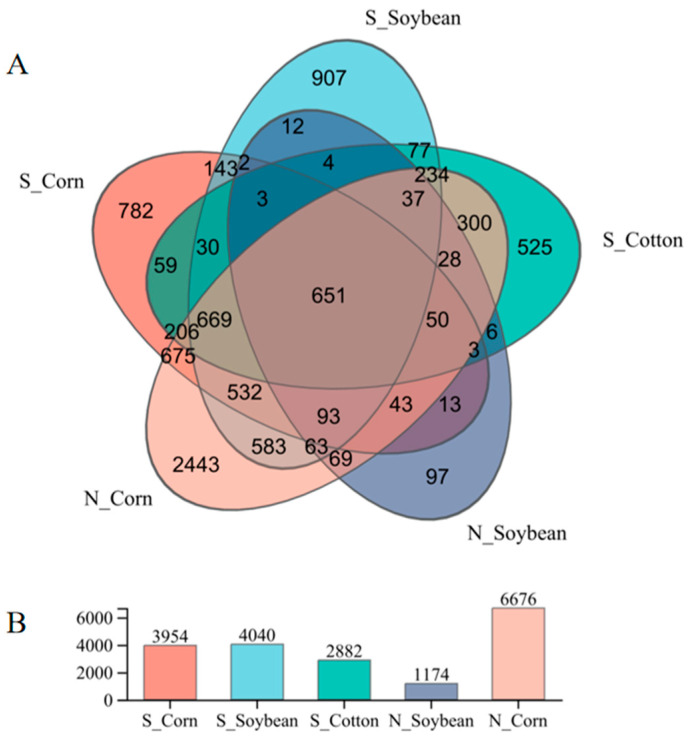
Venn diagram of symbiotic bacteria in different geographical populations of *T. truncatus* in Xinjiang. ((**A**) Note: Different colors denote distinct groups. The central region illustrates the total number of species common to all groups, whereas the peripheral areas represent the species uniquely associated with each individual group. (**B**) The vertical axis of the column chart indicates the number of OTUs (operational taxonomic units) corresponding to the species within each group).

The V3-V4 region of the 16S rRNA gene was sequenced for 190 samples from 17 populations of *T. truncatus* in Xinjiang. The average length of the sequenced fragments was approximately 416 bp. Sequences exhibiting greater than 97% similarity were grouped into operational taxonomic units (OTUs). The Venn diagram illustrating the clustering of effective sequences from all *T. truncatus* samples is presented in Figure 3. A total of 9339 OTUs were identified. Among these, 651 were shared across all 5 populations. In the soybean population, the number of endosymbiotic bacteria OTUs in *T. truncatus* from southern Xinjiang was 3.4 times higher than that in northern Xinjiang. In the corn population, the number of endosymbiotic bacteria OTUs in *T. truncatus* from southern Xinjiang was 1.7 times higher than that in northern Xinjiang (Figure 3).

**Figure 4:**
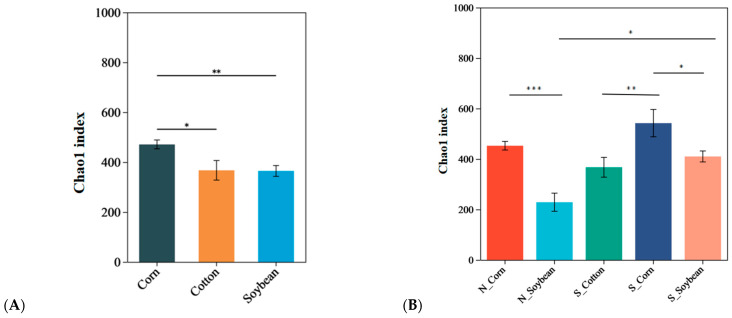
Comparison of symbiotic bacterial community richness in different geographical populations of *T. truncatus* in Xinjiang. (**A**) The Chao 1 index of *T. truncatus* on different hosts. (**B**) The Chao 1 index of *T. truncatus* on different hosts in the northern and southern regions of Xinjiang. (Note: The two groups with differences between the two ends of the upper line are connected; * represents *p* < 0.05, ** represents *p* < 0.01, *** represents *p* < 0.001).

**Figure 5:**
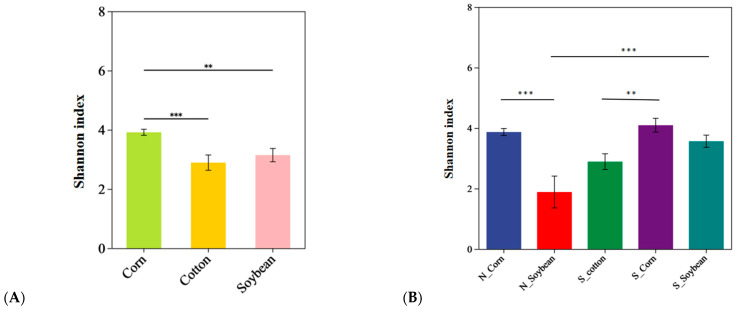
Comparison of symbiotic bacterial community diversity in different geographical populations of *T. truncatus* in Xinjiang. (**A**) The Chao 1 index of *T. truncatus* on different hosts. (**B**) The Chao 1 index of *T. truncatus* on different hosts in the northern and southern regions of Xinjiang. (Note: The two groups with differences between the two ends of the upper line are connected; ** represents *p* < 0.01, *** represents *p* < 0.001).

The alpha diversity analysis for the endosymbiotic bacterial communities of *T. truncatus* from different geographical populations in Xinjiang at the OTU level in Figure 4 and Figure 5. The Chao 1 index and Shannon index are commonly used to estimate species richness and diversity, respectively, within *T. truncatus* populations. In Figure 4, the Chao 1 index of the corn *T. truncatus* population was significantly higher than that of soybean (*p* < 0.01) and cotton populations (*p* < 0.05). In the corn population, no significant difference was observed between southern and northern Xinjiang (*p* > 0.05), whereas in the soybean population, the Chao 1 index in southern Xinjiang was significantly higher than in northern Xinjiang (*p* < 0.05). In Figure 5, the Shannon index of the corn *T. truncatus* population was significantly higher than that of soybean (*p* < 0.01) and cotton populations (*p* < 0.001). In the corn population, no significant difference was observed between southern and northern Xinjiang (*p* > 0.05), whereas in the soybean population, the Shannon index in southern Xinjiang was significantly higher than in northern Xinjiang (*p* < 0.001). The richness and diversity of endosymbiotic bacteria associated with the corn *T. truncatus* population were significantly higher than those in cotton and soybean populations. Furthermore, in the soybean population, both richness and diversity were significantly greater in the southern Xinjiang population compared to the northern Xinjiang population.

### 3.3. Correlation Between Endosymbiotic Bacteria of T. truncatus and Environmental Factors

**Figure 6:**
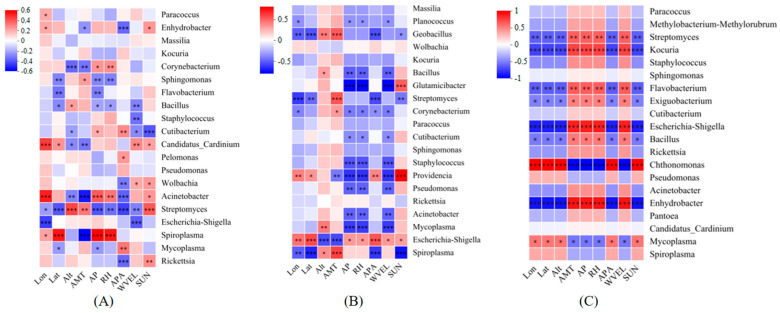
Environmental factor analysis of endosymbiotic bacteria in different geographical populations of *T. truncatus* in Xinjiang (genus level) (Note: (**A**) corn population (118 samples); (**B**) soybean population (48 samples); (**C**) cotton population (24 samples). The asterisks in the color blocks represent significance; * 0.01 < *p* ≤ 0.05, ** 0.001 < *p* ≤ 0.01, *** *p* ≤ 0.001).

At the genus level (Figure 6), the endosymbiotic bacterium *Rickettsia* in *T. truncatus* on corn showed a significant positive correlation with SUN and a significant negative correlation with APA (*p* < 0.05). Additionally, *Spiroplasma* exhibited significant positive correlations with latitude, RH, and AP, while showing a significant negative correlation with AMT (*p* < 0.05). The endosymbiotic bacteria *Spiroplasma* in *T. truncatus* on soybeans exhibited significantly positive correlations with altitude and AMT (*p* < 0.05), while showing significantly negative correlations with longitude, latitude, APA, and SUN (*p* < 0.05). The influence of various environmental factors on *Rickettsia* was not statistically significant (*p* > 0.05). The endosymbiotic bacteria *Spiroplasma* and *Rickettsia* in *T. truncatus* on cotton remained unaffected by environmental changes (*p* > 0.05). In contrast, *Enhydrobacter* and *Chthonomonas* were significantly impacted by environmental factors (*p* < 0.05).

## 4. Discussion

The 16S rRNA sequencing method was employed in this study to investigate the composition and characteristics of the endosymbiotic bacterial community in *T. truncatus* populations from cotton, corn, and soybean plants in both northern and southern Xinjiang. The results elucidated how differences in host plant species and geographical environments influence the diversity of endosymbiotic bacteria in *T. truncatus*. This study revealed that the dominant bacterial phyla associated with *T. truncatus* in different geographical populations in Xinjiang are Pseudomonadota and Bacillota, which aligns with previous research findings. These phyla play crucial roles in regulating its adaptability to various environments [34]. In this study, *Wolbachia*, *Cardinium*, *Spiroplasma*, and *Rickettsia* were found to be widely present in *T. truncatus* populations in Xinjiang. Consistent with previous reports, these four bacteria are commonly detected across various strains of spider mite [35]. However, prior studies have not reported any instances of *Rickettsia* infection in *T. truncatus*, including in Korla, Xinjiang [36]. In contrast, the current study revealed a remarkably high infection rate of 92.8% for *Rickettsia* in *T. truncatus*. This may be attributed to the fact that the sample points investigated in this study did not encompass this specific area, resulting in regional disparities and consequently inconsistent findings. Some studies have demonstrated that *Bemisia tabaci* infected with *Rickettsia* can upregulate the expression of heat-tolerance-related genes, leading to a substantial enhancement in their ability to withstand heat shock [37]. Therefore, the high expression of *Rickettsia* in *T. truncatus* in Xinjiang might represent a specific adaptive response to the unique environmental conditions of the area. Nevertheless, the precise role of this bacterium in *T. truncatus* remains to be further elucidated through additional research.

The host plant plays a crucial role in shaping the structure and diversity of symbiotic bacteria within insects [38]. In this study, the diversity of endosymbiotic bacteria in *T. truncatus* on corn was found to be 3.4 times and 1.8 times higher than that on cotton and soybean. Among the 17 surveyed sampling sites, this study identified 11 populations of the corn *T. truncatus*, distributed across both northern and southern regions. In contrast, cotton populations were detected at only two sites, both located in the southern region. Secondly, the dominant pest mite species on cotton in the northern region is the *Tetranychus turkestani*, which may contribute to the relatively low diversity of endosymbiotic bacteria observed in cotton *T. truncatus* populations. Furthermore, soybean cultivation in Xinjiang remains limited compared to other crops. The broader geographic distribution and higher sampling frequency of corn populations may contribute to the greater diversity of endosymbiotic bacteria observed in *T. truncatus* compared to those from cotton and soybean. Furthermore, the nutrient contents in the leaves of different host plants, including soluble sugar, soluble protein, and amino acids, as well as the chemical defense compounds produced upon insect feeding, can significantly influence the composition of endosymbiotic bacterial communities [39]. The secondary metabolites produced by different host plants exhibit distinct profiles. For instance, benzoxazine compounds in corn, gossypol and tannic acid in cotton, and isoflavone compounds in soybeans all possess bactericidal properties to varying extents [40,41,42,43]. These substances may differentially affect the endosymbiotic bacterial community of *T. truncatus*. In addition, the microenvironmental conditions on the leaf surfaces of different host plants, including variations in nutrient composition and pH levels, may also contribute to the differences observed in the composition of their endosymbiotic bacterial communities [14]. In this study, the composition of endosymbiotic bacteria in *T. truncatus* significantly among three host plants—cotton, corn, and soybean—at the genus level. *Spiroplasma* and *Cardinium* were more abundant in *T. truncatus* associated with cotton. In contrast, *Spiroplasma* was the dominant bacterial genus in *T. truncatus* on soybean, whereas *Rickettsia* predominated in *T. truncatus* on corn. As previously reported in earlier studies, the host species can influence the composition of dominant endosymbiotic bacteria. For example, *Wolbachia* and *Spiroplasma* were identified as dominant endosymbiotic bacteria in tomatoes, whereas their relative abundance decreased markedly when corn served as the host plant [30]. Furthermore, co-infection with *Spiroplasma* and *Cardinium* was found to enhance the fecundity and development of *T. truncatus* [30]. The reproductive rate of adult female *Typhlodromus occidentalis* infected with *Cardinium* increased by approximately 50% [44]. In this study, the concurrent presence of these two bacterial symbionts on cotton and soybean plants may contribute to enhanced adaptability of *T. truncatus*.

The composition of endosymbiotic bacteria in *T. truncatus* exhibits variation between different geographical populations in the Xinjiang region. The diversity of endosymbiotic bacteria in *T. truncatus* on the same host was higher in southern Xinjiang than in northern Xinjiang. Specifically, the Chao1 index for *T. truncatus* on soybeans in southern Xinjiang was 1.8 times higher than that in northern Xinjiang, while the Chao1 index for *T. truncatus* on corn was 1.2 times higher in southern Xinjiang compared to northern Xinjiang. This may be attributed to the high-temperature and low-humidity in southern Xinjiang. A previous study has shown that the high temperature of Yangtze River Basin exhibited the highest bacterial diversity of Aphis gossypii, followed by the Northwestern Inland Region, and then the Yellow River Basin [45]. In this study, the relative abundance of Bacillota in *T. truncatus* in southern Xinjiang was approximately 1.6 times higher than that in northern Xinjiang. Previous studies have demonstrated that under high-temperature stress, the relative abundance of Bacillota in both *Drosophila melanogaster* and *Porcellio scaber* increases to facilitate adaptation [46,47]. Therefore, the higher relative abundance of Bacillota in *T. truncatus* in southern Xinjiang than that in northern Xinjiang is potentially attributable to the higher temperatures in the south. Additionally, the relative abundance of *Rickettsia* in *T. truncatus* on corn is greater in southern Xinjiang than in northern Xinjiang, as is relative abundance of *Spiroplasma* in *T. truncatus* on soybean. Under high-temperature conditions, the relative abundances of *Rickettsia* in *Bemisia tabaci* and *Spiroplasma* in aphids increase [48]. Both bacteria are capable of enhancing the high-temperature tolerance of their respective insect hosts [36]. Furthermore, agricultural practices and crop planting patterns in Xinjiang may influence the occurrence and distribution of *T. truncatus*, thereby contributing to the uneven sample sizes observed in this study. During the field sampling, it was noted that soybean cultivation was more prevalent in the southern region compared to the northern region of Xinjiang. Such regional differences in soybean planting density may account for the variations in both the number of *T. truncatus* samples and the composition of their symbiotic microbial communities between the northern and southern regions.

Although the *T. truncatus* is present in both northern and southern Xinjiang, the diversity of its endosymbiotic bacteria on the same host species is greater in the southern region compared to the northern region. Furthermore, the diversity of endosymbiotic bacteria associated with the *T. truncatus* is higher on corn than on cotton or soybeans.

## Figures and Tables

**Table 1:** The geographical coordinates of the collection sites, hosts, and climate parameters of *T. truncatus* in Xinjiang.

Regional Distribution	Sampling Location	Host	Host Variety	Sample Size	Population Code	Lon (°E)	Lat (°N)	Alt (m)	AMT (°C)	AP (mm)	RH (%)	APA (hPa)	WVEL (m/s)	SUN (h)
Northern Xinjiang	Kekedala City	Soybean	Xindadou 26	12	N_68D	80.624	44.127	578	12.90	241.51	42.68	934.81	3.57	4797.40
	Shuanghe City	Corn	Xinyu 97	9	N_81Y	82.485	44.772	270	11.36	149.61	44.23	961.04	2.42	4776.80
	Shuanghe City	Corn	Xinyu 97	12	N_82Y	82.592	44.782	636	11.36	149.61	44.23	961.04	2.42	4776.80
	Shihezi City	Corn	Huamei 1	12	N_121Y	86.048	44.291	493	10.96	172.4	45.38	964.71	2.98	4776.00
	Huyanghe City	Corn	Huaxi 703	6	N_124Y	84.874	44.796	284	10.38	177.76	45.24	948.90	3.11	4799.00
	Habahe County, Altay Prefecture	Corn	Heyu 187	12	N_AHY	86.480	48.085	512	3.10	331.78	56.56	887.88	3.12	4812.80
	Urumqi City	Corn	Bixiang 101	12	N_ANY	87.505	43.982	566	10.96	225.40	44.19	933.36	2.97	4774.20
	Wusu City, Tacheng Prefecture	Corn	Denghai 550	12	N_GY	84.303	44.413	483	6.43	203.60	49.68	872.90	1.85	4828.40
	Shanshan County, Turpan City	Corn	Sitai 159	12	N_SY	90.546	43.005	517	11.98	25.89	30.51	908.67	3.24	4805.60
	Fukang City, Changji Hui Autonomous Prefecture	Corn	Sitai 112	7	N_JY	88.076	44.170	560	9.59	189.26	44.36	928.99	2.90	4782.40
Southern Xinjiang	Aksu City, Aksu Prefecture	Cotton	Xinluzhong 84	12	S_AMr	80.263	41.168	1162	13.58	57.37	31.85	894.88	2.69	4832.60
	Aksu City, Aksu Prefecture	Soybean	Fengchan 80	12	S_LDr	80.263	41.168	1162	13.58	57.37	31.85	894.88	2.69	4832.60
	Xinhe County, Aksu Prefecture	Corn	Zhengdan 958	12	S_XY	82.577	41.546	1017	12.79	75.75	35.18	892.17	2.9	4821.20
	Tumushuke City	Corn	Xianyu 335	12	S_4414Y	79.133	39.827	1164	13.64	88.23	33.53	892.17	2.91	4779.40
	Tumushuke City	Soybean	Zhonghuang 35	12	S_44D	79.133	39.827	1164	13.64	88.23	33.53	892.17	2.91	4779.40
	Tumushuke City	Soybean	Heihe 11	12	S_53D	79.089	39.868	1098	13.64	88.23	33.53	892.17	2.91	4779.40
	Tumushuke City	Cotton	Tahe 2	12	S_53M	79.089	39.868	1098	13.64	88.23	33.53	892.17	2.91	4779.40

Note: Lat: latitude; Lon: longitude; AMT: annual mean temperature; Alt: altitude; AP: annual precipitation; RH: relative humidity; APA: average pressure; WVEL: wind velocity; SUN: annual sunshine duration.

## Data Availability

The original data presented in the study are openly available in online repositories at NCBI-PRJNA1305143.
